# Perspective: Quality Versus Quantity; Is It Important to Assess the Role of Enhancers in Complex Disease from an In Vivo Perspective?

**DOI:** 10.3390/ijms21217856

**Published:** 2020-10-23

**Authors:** Andrew R. McEwan, Alasdair MacKenzie

**Affiliations:** School of Medicine, Medical Sciences and Nutrition, Institute of Medical Sciences, Foresterhill, University of Aberdeen, Aberdeen AB25 2ZD, UK; a.r.mcewan@abdn.ac.uk

**Keywords:** complex disease, gene regulation, chromatin modification, comparative genomics, CRISPR genome editing, promoter, enhancer, polymorphisms, mental health, alcohol abuse, anxiety, pharmacogenomics

## Abstract

Sequencing of the human genome has permitted the development of genome-wide association studies (GWAS) to analyze the genetics of a number of complex disorders such as depression, anxiety and substance abuse. Thanks to their ability to analyze huge cohort sizes, these studies have successfully identified thousands of loci associated with a broad spectrum of complex diseases. Disconcertingly, the majority of these GWAS hits occur in non-coding regions of the genome, much of which controls the cell-type-specific expression of genes essential to health. In contrast to gene coding sequences, it is a challenge to understand the function of this non-coding regulatory genome using conventional biochemical techniques in cell lines. The current commentary scrutinizes the field of complex genetics from the standpoint of the large-scale whole-genome functional analysis of the promoters and cis-regulatory elements using chromatin markers. We contrast these large scale quantitative techniques against comparative genomics and in vivo analyses including CRISPR/CAS9 genome editing to determine the functional characteristics of these elements and to understand how polymorphic variation and epigenetic changes within these elements might contribute to complex disease and drug response. Most importantly, we suggest that, although the role of chromatin markers will continue to be important in identifying and characterizing enhancers, more emphasis must be placed on their analysis in relevant in-vivo models that take account of the appropriate cell-type-specific roles of these elements. It is hoped that offering these insights might refocus progress in analyzing the data tsunami of non-coding GWAS and whole-genome sequencing “hits” that threatens to overwhelm progress in the field.

## 1. Introduction

Genome-wide association studies (GWAS) have revolutionized the genetic analysis of human disease. Ever since the sequencing of the human genome and the development of gene chip technologies, GWAS has been able to scan hundreds of thousands of genetic variants in tens or even hundreds of thousands of patients to deliver a greater understanding of the genetic architecture of complex human disease. Thanks to GWAS, the number of genetic loci with significant association with complex disease (*p* < 1 × 10^−6^), known as GWAS “hits”, now number in the thousands [1]. One promising use of the data generated by these GWAS studies is in the calculation of polygenic risk scores that, by adding together the disease risk of many individual alleles, seek to predict the likelihood of developing a complex disease [2]. From the standpoint of functionality, when a GWAS “hit” occurs within a gene coding sequence we can use a plethora of well-understood biochemical methodologies to understand the molecular mechanisms affected by a specific allelic variant. Frustratingly, however, the vast majority of these GWAS hits do not occur neatly in coding regions but within the 98% of the genome that does not encode protein; a region of the genome referred to as the non-coding genome [3]. Another more recent and widely focused study further confirmed that the majority of GWAS hits were non-coding but also made the somewhat bewildering prediction that complex human diseases are probably influenced by the combined input of many hundreds if not thousands of genetic variants [4]. This study further recommended that a much better understanding of cell-type-specific regulatory networks and the regulatory genome must be achieved before an understanding of the genetic basis of complex disease can be gained [4].

### What Is the Regulatory Genome?

Most scientists with a passing knowledge of molecular genetics are aware of the existence of sequences adjacent to the transcriptional start sites of genes called promotors [5]. These act to attract and orientate the pre-initiation complex (PIC) to the transcriptional start site (TSS) of genes [6]. The PIC comprises a complex of proteins that include RNA polymerase II (RNApolII) [7], which is now known to initiate transcription from promoters bidirectionally [8]. Attempts have been made to classify promoters based on their tissue specificity, interaction with histones, the relative integrity of their TSSs and the histone methylation signatures (triple methylation of lysine 4 of histone 3; H3K4me3) associated with their activity [9]. More recent Drosophila-based studies stratified promoters according to the absence or presence of TATA box or DPE sequences [10]. Promoter regions are often associated with regions of DNA called CpG islands that contain a much higher proportion of CpG dinucleotides than the surrounding genome. These CpGs act as a target of the DNA methyltransferase (DNMT) enzyme, which methylate’s the cytosine within the CpG dinucleotide to form 5-methyl cytosine (5mC) [11]. Changes in the methylation of CpG islands have been associated with disease states such as alcohol and drug addiction [12], and altered CpG methylation has a significant effect of levels of promoter activity [13]. Critically, many promoters rely on the proximity of other elements, such as enhancers, to drive their activity in specific cells and tissues [14]. It was estimated by the ENCODE consortium that these enigmatic and poorly understood enhancer sequences make up as much as 10% of the human genome, five times greater than the coding genome, and may comprise over 2 million enhancers [15]. However, in light of the increasing evidence for a role of non-coding regulatory elements in susceptibility to complex disease, there are concerns that our limited ability to detect and functional characterize cell-type-specific enhancers threatens to derail efforts to understand the molecular basis of complex disease and to produce potential personalized therapies. So what are enhancers?

## 2. Identifying Enhancer Sequences; Lessons from Developmental Biology

Classically, an enhancer is defined as a region of non-coding DNA that is required to increase the activity of a promoter region. In contrast to promoter regions, enhancers are also defined as being orientation- and distance- independent [16]. From an evolutionary perspective, it is instructive that even the most complex single-cell organisms lack enhancer sequences [17]. Indeed, there is an emerging consensus that enhancers evolved to allow cells to communicate with each other to build complex multicellular systems and to organize cell division and differentiation during the embryonic development of the first multicellular organisms [17].

Although most complex diseases affect adult humans, many of the lesions leading to disease may be developmental in origin, and it is through developmental biology that we have received our most revealing insights into enhancer biology. For example, pivotal studies on the causes of human developmental disorders such as preaxial–polydactyly (PPD) [18] and Pierre Robin sequence (PRS) [19] have emphasized the huge genomic distances (1.5 and 1Mb respectively) over which many enhancers affect the activity of the promoters they regulate. Both of these studies also emphasize the extreme context-dependency of many enhancer sequences. Thus, the activity of the PRS enhancer sequences studied by the Wysocka lab was only revealed in cranial neural crest cells and could not be detected in stem cells [19]. These studies highlight that enhancers are required to allow genomes of multicellular organisms to communicate during their embryonic development and, most importantly, to respond appropriately within the correct context and to the appropriate cues [20]. In this regard, enhancers are like the “sense organs” of the genome in that they detect whether a specific cell–cell interaction or environmental response has triggered a particular set of signal transduction cascades [21]. These signal transduction cascades then trigger transcription factor proteins to bind enhancers and the identity of these transcription factors, and their precise affinity for their target sequence combined with the syntax of their binding, largely determines the precise transcriptional response of a given enhancer [22,23,24]. Although we still need to define the limits to the range at which enhancers can influence promoter activity, it is clear that not all enhancers are able to interact with all promoters [25]. The range and choice of promoters that enhancers influence is dependent on factors such as enhancer–promoter specificity and whether they are separated by a third type of regulatory element called an insulator [26]. Insulator sequences, often referred to as TAD boundary elements, operate in combination with CTCF and cohesin proteins and play a role in dividing the genome into topological associating domains or TADs [27]. It has been suggested that TADs limit the influence of enhancers on other promoters [28], although a full understanding of the blocking effects of insulators/TAD boundary domains remains to be determined [29]. In addition to their identification through CTCF binding, TADs can be identified using chromatin conformation capture techniques, such as 5C and HiC, which are able to detect long-distance chromatin interactions [30]. Although we are still to fully understand the mechanisms modulating these interactions, it is evident that loops of DNA formed by the threading of chromatin through cohesin protein rings [27], interact within discrete but transient regions of the nucleus called chromatin hubs (previously referred to as transcription factories) [31]. It has also been posited that much of the long non-coding RNA (lncRNA) found in cells may derive from the active hauling of chromatin by tethered RNApolII into these hubs [32]. This hypothesis is further supported by the observation that regions of DNA on either side of active enhancers are transcribed by RNApolII [33]. Nevertheless, before we can understand how enhancers work in maintaining health, and how polymorphisms and epigenetic factors influence their activity, we must be able to reliably identify and functionally characterize them.

## 3. Chromatin Markers

**Figure 1:**
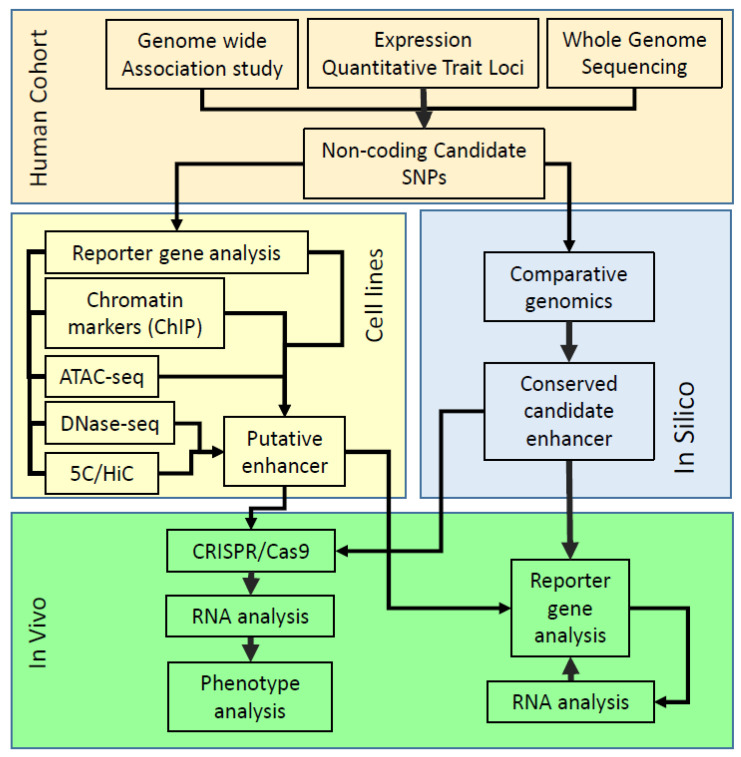
Simplified flow diagram demonstrating the relationship between different techniques designed to identify and characterise the functional consequences of disease-associated non-coding polymorphisms on enhancer activity. CRISPR/Cas9 could be used to functionally dissect enhancers or, using homologous recombination, reproduce disease-associated alleles. Reporter gene analysis could reflect the quantitative (cell line) or qualitative (in vivo) consequences of a disease-associated polymorphism on the functioning of a conserved or species-specific enhancer.

As previously highlighted, most of the major insights into identifying enhancer elements and an appreciation of the importance of their biology have come from developmental biology. It could be argued that other fields, which study the biology of complex human disease, lag behind in terms of their identification and characterization of cell-type-specific enhancer regions that GWAS suggest are important in the development of these diseases. Are there lessons that these fields can learn from developmental biology? Thanks to efforts by developmental biologists, several histone modifications and the interaction of certain co-factors have become widely accepted markers of active enhancers. These include histone 3, which has a mono-methylated fourth lysine residue (H3K4me1) and histone 3 acetylated on lysine 27 (H3K27Ac) [34]. These histone marks are detected using chromatin-immunoprecipitation (ChIP) technologies that use antibodies raised against each modified histone (Figure 1) [34]. The p300 co-factor, also detected using ChIP, another accepted marker of enhancer activity and acts as a histone acetyl transferase (HAT), whose activity is responsible for generating H3K27ac [35]. Furthermore, regions of chromatin that are transcriptionally active are often found in an “open” configuration, where the DNA and histones making up the chromatin associate more loosely. This “open” DNA can be detected using techniques such as DNAse-seq, which depends on a DNAse enzyme gaining access to the DNA (Figure 1) [36], or the more recently developed and amenable ATAC-seq that uses the Tn5 transposase to ligate “barcode” primers to open chromatin to aid subsequent next generation sequencing(Figure 1) [37]. Yet another technique that has been used to detect active enhancers is a technique called Cap Analysis of Gene Expression (CAGE) that detects enhancer activity by virtue of their bidirectional transcription [33]. The development of these methods of identifying active markers has hugely accelerated our ability to functionally annotate the non-coding genome. However, the extreme context-dependency demonstrated by many enhancers ensures that finding the appropriate cellular context in which these enhancers are active, and thus open to detection by the methods described above, is often problematic. This problem was effectively illustrated through the study of enhancers that drive the expression of the *SOX9* gene in the lower jaw, where enhancer activity (H3K27ac, H3K4me1 and p300) could not be detected in stem cells but only within primary embryonic cranial neural crest cells [19], demonstrating the strong context-dependency of these enhancers.

The issue of context-dependency is one that somewhat decreases the impact of the ENCODE data in identifying context-dependent or cell-type-specific enhancers. Released with much fanfare in 2012, the data generated by ENCODE depended mainly on a combination of high-throughput next-generation sequencing and homogeneous cell culture in a large number of different human cell lines [15]. Despite its undoubted success in terms of numbers of publications in high-profile journals, the ENCODE data release was greeted by a fair degree of skepticism [38]. One of the most contentious issues was the suggestion by ENCODE that regulatory regions were not well conserved [15]. The inference was that enhancer regions evolved rapidly in each species, a hypothesis that was later supported by a study based on the comparison of enhancer markers (H3K27ac) and conservation focusing on primary hepatocytes [39]. A further p300/CBP co-factor analysis in adult and foetal human heart tissues also concluded that vertebrate enhancers evolved rapidly and were not well conserved between species [40,41]. The conclusions of these studies were controversial as they appeared to fly in the face of evolutionary theory [38]. Although histone methylation/acetylation, p300 and open chromatin (DNase1 sensitivity and ATAC) are now widely accepted as proxies for active enhancer regions, questions need to be asked regarding the relevance and suitability of the cell types used in many of these studies. Thus, whilst it is likely that H3K27ac, H3K4me1 and p300 enhancer marks will remain the gold standard in detecting enhancers, their relevance in identifying and characterizing enhancers will only be as good as the cell lines used. One possible direction we can use to partly address this concern is to identify enhancer by virtue of their conservation. For example, high levels of sequence conservation are associated with both the Shh enhancer [18] and Pierre Robin sequence PRS enhancer [19]. Another exemplary study of GWAS-associated SNPs associated with neuroblastoma succeeded in identifying an SNP (rs2168101 G > T) within a highly conserved enhancer inside intron 1 of the *LMO1* gene [42]. Taken together, it is clear that the majority of studies that have successfully identified functional enhancer regions in higher vertebrates have also identified extensive conservation in these regions.

## 4. An Evolutionary Perspective

The logic behind comparative genomics is based on the hypothesis that genomic sequences critical to species survival change at a much slower rate through evolutionary time [43,44]. Prior to the discovery and widespread use of active enhancer proxies such as p300, H3K4me1 and H3K27ac, a number of studies had successfully identified cell-type-specific activity in the majority of conserved regions identified using transgenic embryos [44]. The identification of these sequences in human DNA involves the computer alignment of hundreds of vertebrate genome sequences available online through portals such as ENCODE, EMSEMBL and the UCSC browser [43,44]. We also found that proven cell-type-specific enhancers controlling the *Msx1* gene, identified through painstaking functional dissection and analysis in transgenic embryos [45], were highly conserved once comparison of genome sequences of mice, humans and chickens became possible [46,47]. Moreover, intuitively, it makes little sense that the complex syntax of transcription factor binding sites, that are essential for the function of enhancers that support these complex cell-type-specific patterns displayed by many genes, should not also be conserved to some degree [48]. This was further supported by the observation that nearly 10% of the human genome is under selection and has been conserved during evolution despite only 1.7% of the genome encoding proteins [49]. Whilst there is evidence of increased plasticity in the evolution of the non-coding genome compared to the coding genome that can account for much of the observable difference between different species and between individuals within a species [50], we will present further evidence that there is a clear case for including comparative genomics in looking for functional disease-associated enhancer sequences in the human genome (Figure 1).

## 5. Conserved Enhancers in Adult Brain Activity

In addition to our embryonic development, many aspects of adult human health are also highly dependent on tightly controlled cell-type-specific gene regulation. For example, human health and homeostasis are tightly regulated by a myriad of different neuropeptides that need to be expressed in precise regions of the hypothalamus, amygdala and sensory neurons to modulate appropriate levels of food intake, mood and pain perception [51,52]. Thus, identification of the enhancer regions that control the cell-type-specific expression of these neuropeptides, and how they might be affected by polymorphic variation, will be critical to our understanding of health and disease. For example, substance-P (SP; encoded by *TAC1* gene) is a neuropeptide expressed in c-fibre sensory neurons and the medial amygdala where it is critical to pain perception and mood modulation [53,54]. We first used comparative genomics in species as diverse as mammals and birds to identify a highly conserved enhancer that lay 158kb from the *TAC1* gene, which drove reporter gene expression in SP expressing amygdala neurons [55]. Later transgenic analysis of an even remoter conserved enhancer (214kb from *TAC1* gene) demonstrated its ability to not only drive reporter expression in SP expressing C-fibre sensory neurons but to also switch reporter gene expression to larger diameter A-fibres in response to inflammatory stimuli—an expression of SP associated with hyperalgesia [56,57,58].

We also used comparative genomics to explore the regulation of another neuropeptide; galanin (encoded by the *GAL* gene) that played a role in mood modulation as well as preference for fat and ethanol [51,59]. We identified a region of highly conserved DNA (GAL5.1) lying 42kb from the *GAL* gene that we cloned into a reporter plasmid. Following microinjection of this plasmid into the pronucleus of a mouse embryo and subsequent oviduct transfer (Figure 1) we found that GAL5.1 drove expression of the reporter gene in *Gal* expressing cells of the hypothalamus and amygdala of resulting transgenic mice [60]. We also used CRISPR/CAS9 genome editing technologies (Figure 1) to delete GAL5.1 from the mouse genome. This was achieved by injecting CAS9 mRNA and guideRNA (gRNA), designed to direct the deletion of the GAL5.1 enhancer, into the cytoplasm of one-cell mouse embryos. Following oviduct transfer and birth of these mice, we found a high proportion lacked the GAL5.1 enhancer (Figure 1). Quantitative reverse transcriptase analysis of these lines showed a major reduction in *Gal* expression in the amygdala and hypothalamus. In addition, these animals demonstrated significantly reduced fat and alcohol consumption and reduced anxiety-like behavior in males [61]. Quantitative analysis of two different allelic variants of the human GAL5.1 enhancer identified significant differences in the activity of these variants in primary hypothalamic cells [60]. Most surprisingly, a parallel study of a large human cohort (UK Biobank) succeeded in identifying an association between increased anxiety and ethanol consumption in males carrying the stronger GAL5.1 allele [61]. Intriguingly, we were unable to identify chromatin markers such as H3K4me1, H3K27ac or DNAse1 sensitivity within any of the enhancer that we have studied over the years using available online databases such as the ENCODE consortium. The likely reason behind this observation is that, because of the extreme tissue specificity of the enhancers we analyzed, they are inactive within the cell types available to ENCODE and would, therefore, lack the histone markers, p300 and DNase sensitivity marks diagnostic of active enhancers.

The next logical step in the in vivo analysis of enhancer function will be to reproduce the different human disease-associated allelic variants of each enhancer in mice and analyze their effects on the physiology and behavior of the resultant animals. This would likely require the humanization of entire enhancer regions with either allelic variant and a comparison of the behavior of the different lines. CRISPR-driven humanization of large regions of the mouse genome represents a challenging process that, in addition to CAS9 and gRNA, requires the co-injection of repair template DNA, designed to trigger the cells’ homology-directed repair pathway, into the nucleus of a 1-cell mouse zygote. Whilst this approach has been successfully used to humanize gene coding regions in mice [62,63,64], it has yet to be used to humanize enhancer regions. The alternative, and more conventional, strategy to humanization involves embryonic stem (ES)-cell targeting and blastocyst microinjection [65]. However, ES-cell targeting is an order of magnitude more expensive and time-consuming than CRISPR-based options thus placing it outside the financial constraints of most small labs.

## 6. Gene Regulation and Pharmacogenomics?

Despite major efforts to identify novel drug targets within the coding regions of the genome, little or no attention has been paid to the possible role of the non-coding genome in altering drug response or even as a drug target in its own right. In the past, a number of GWAS studies have identified that, in addition to disease, the non-coding genome also acts as a reservoir of drug stratification loci that alter the response to drugs between individuals within the population [66]. In an attempt to identify drug stratification loci within the genome, we used comparative genomics to explore the regulation of the gene (*CNR1*) encoding the cannabinoid-1 receptor (CB_1_). The pharmacogenomics of CB_1_ has gained considerable interest following the legalization of therapeutic cannabinoids in many countries around the world, including the legalization of recreational cannabis use in a number of states in the US [67]. We used comparative genomics to identify a polymorphic enhancer region within intron1 of the CNR1 locus that lay in strong linkage disequilibrium (LD; a phenomenon where groups of alleles travel together in a population) with a group of SNPs that had been previously associated with addictive behaviors [68]. Initial *in-vitro* analysis of this enhancer region showed considerable differences in the enhancer activity of allelic variants [68]. As for GAL5.1, we used CRISPR genome editing to delete this enhancer from the mouse genome but only achieved a relatively modest, although significant, decrease in the hippocampal expression of the *Cnr1* gene in these animals, suggesting regulatory redundancy [69]. Nevertheless, behavioral analysis of these animals demonstrated a significant difference in their response to CB_1_ agonism and alcohol intake [69], suggesting that despite its modest effects on *CNR1* expression, the *Cnr1*-intron 1 enhancer plays a role in CB_1_ function that has been conserved for hundreds of millions of years. Taken together, these experiments suggest a future direction for the analysis of the non-coding genome in the burgeoning field of pharmacogenetics.

## 7. Enhancers as Future Personalised Drug Targets?

Although a great deal is known of the interaction of enhancers and transcription factors, almost nothing is known of the signal transduction pathways that influence these interactions [21]. In order to identify the signal transduction pathways that influence the activity of the GAL5.1 enhancer, we used a series of agonists and antagonist to identify the involvement of protein kinase C (PKC) pathways in stimulating GAL5.1 activity whilst ruling out the involvement of protein kinase A (PKA) or the MAPkinase pathways [60,61]. Critically, we were able to demonstrate that the allelic variant of GAL5.1, associated with increase ethanol intake and anxiety, responded more strongly to PKC activation than did the protective minor allele [60,61]. In another study, we observed that the C-allele of a repressor region (rs12273363), associated with mood disorders, modulated the activity of BDNF promoter 4 in an allele-specific manner following cell depolarization or the combined activity of PKA and PKC pathways [70]. These studies lay the groundwork for developing a better understanding of the “druggable” signal transduction pathways in the cell that control enhancer action, which may eventually give us clues for the development of future personalised therapeutics.

## 8. Nature Versus Nurture: Epigenetics and the Functional Non-Coding Genome

CpG methylation is an epigenetic marker that is strongly affected by environmental factors such as diet, early life stress, or the life-styles led by our parents [71]. Environmentally altered CpG methylation, in turn, can modulate susceptibility to diseases such as depression, addiction and anxiety in subsequent generations [72,73]. Although the effects of epigenetic modification of promoter regions through CpG methylation is well established [73,74] there is also evidence that methylation of enhancer regions affects their activity [75,76]. For example, decreased methylation of an enhancer responsible for expression of the AgRP gene in the amygdala and hypothalamus was found to result from early life stress (maternal separation). The results of this reduction in AgRP enhancer methylation was an increase in AgRP gene expression and an increase in anxiety and depression-like behavior in mice subjected to maternal separation [77]. Most intriguingly, allelic variation can often introduce or delete CpG dinucleotides within functional regulatory regions, rendering these enhancers more or less susceptible to the effects of CpG methylation. For example, the GAL5.1 enhancer contains two loci that modulate its cell-type-specific activity such that the GG haplotype has one more CpG than the rarer CA haplotype [60,61]. Moreover, the enhancer within *CNR1* intron 2 contains an SNP whose disease-associated T-allele removes a CpG [78,79]. From these preliminary observations, we can already see that polymorphic enhancer and promoter regions may serve as regions of the genome where genetics and environment interact to ensure health or to exacerbate disease susceptibility.

## 9. Conclusions

A direction for the future understanding of the role of the non-coding genome in complex disease has been presented. Thanks to its decreasing costs and increasing efficiency, the use of next-generation sequencing (NGS) to produce whole-genome sequencing (WGS) technologies to sequence the genomes of large human disease cohorts is poised to supersede GWAS as the method of choice for identifying allelic variants associated with complex disease [80,81]. Another very promising direction in determining the functional consequences of non-coding allelic variation is the analysis of expression quantitative trait locus (eQTL) data as typified by the GTEx consortium [82]. This approach is based on the statistical relationship between allelic variants and levels of gene expression on a multi-tissue–whole-genome level. Recent successful use of eQTL analysis was able to detect regulatory variants that conferred risk to ADHD, schizophrenia and bipolar disorder in prenatal brain tissues [83]. There are concerns, however, that the cascade of data coming from GWAS and eQTL analysis will become a roaring deluge once WGS replaces GWAS as a routine way to analyze human disease cohorts.

Although there will inevitably be regions of the genome associated with human disease that are unique to humans, it is likely that the majority of disease-causing variants will be in conserved functional regions of the non-coding genome that control physiologies and behaviors common to all mammals [50]. Another advantage to initially studying conserved regions is the ability to model the phenotypic effects of modifying enhancer regions common to both species (humans and mice), which cannot be done for non-conserved sequences. Gaining an understanding of the genomic architecture of human disease can only be built on the foundations of a robust functional annotation of the conserved non-coding genome using in-vivo models. Most importantly, developing an understanding of enhancers in health, and their role in disease, will require a root and branch re-education of the nascent scientific community so that the next generation of scientists are ready for the challenges ahead.

## References

[1] Uffelmann E., Posthuma D. (2020). Emerging Methods and Resources for Biological Interrogation of Neuropsychiatric Polygenic-Signal. Biol. Psychiatry.

[2] Mallet J., le Strat Y., Dubertret C., Gorwood P. (2020). Polygenic Risk Scores Shed Light on the Relationship between Schizophrenia and Cognitive Functioning: Review and Meta-Analysis. J. Clin. Med..

[3] Hindorff L.A., Sethupathy P., Junkins H.A., Ramos E.M., Mehta J.P., Collins F.S., Manolio T.A. (2009). Potential etiologic and functional implications of genome-wide association loci for human diseases and traits. Proc. Natl. Acad. Sci. USA.

[4] Boyle E.A., Li Y.I., Pritchard J.K. (2017). An Expanded View of Complex Traits: From Polygenic to Omnigenic. Cell.

[5] Danino Y.M., Even D., Ideses D., Juven-Gershon T. (2015). The core promoter: At the heart of gene expression. Biochim. Biophys. Acta.

[6] Juven-Gershon T., Kadonaga J.T. (2009). Regulation of gene expression via the core promoter and the basal transcriptional machinery. Dev. Biol..

[7] Davidson S., Macpherson N., Mitchell J.A. (2013). Nuclear organization of RNA polymerase II transcription. Biochem. Cell Biol..

[8] Orekhova A.S., Rubtsov P.M. (2013). Bidirectional promoters in the transcription of mammalian genomes. Biochemistry.

[9] Carninci P., Sandelin A., Lenhard B., Katayama S., Shimokawa K., Ponjavic J., Semple C.A., Taylor M.S., Engstrom P.G., Frith M.C. (2006). Genome-wide analysis of mammalian promoter architecture and evolution. Nat. Genet..

[10] Natsume-Kitatani Y., Mamitsuka H. (2016). Classification of Promoters Based on the Combination of Core Promoter Elements Exhibits Different Histone Modification Patterns. PLoS ONE.

[11] Du X., Han L., Guo A.Y., Zhao Z. (2012). Features of methylation and gene expression in the promoter-associated CpG islands using human methylome data. Comp. Funct. Genom..

[12] Mahna D., Puri S., Sharma S. (2018). DNA methylation signatures: Biomarkers of drug and alcohol abuse. Mutat. Res..

[13] Jeziorska D.M., Murray R.J.S., de Gobbi M., Gaentzsch R., Garrick D., Ayyub H., Chen T., Li E., Telenius J., Lynch M. (2017). DNA methylation of intragenic CpG islands depends on their transcriptional activity during differentiation and disease. Proc. Natl. Acad. Sci. USA.

[14] Chepelev I., Wei G., Wangsa D., Tang Q., Zhao K. (2012). Characterization of genome-wide enhancer-promoter interactions reveals co-expression of interacting genes and modes of higher order chromatin organization. Cell Res..

[15] Dunham I., Kundaje A., Aldred S.F., Collins P.J., Davis C.A., Doyle F., Epstein C.B., Frietze S., Harrow J., Kaul R. (2012). An integrated encyclopedia of DNA elements in the human genome. Nature.

[16] Benoist C., Chambon P. (1981). In vivo sequence requirements of the SV40 early promotor region. Nature.

[17] Sebe-Pedros A., Ballare C., Parra-Acero H., Chiva C., Tena J.J., Sabido E., Gomez-Skarmeta J.L., di Croce L., Ruiz-Trillo I. (2016). The Dynamic Regulatory Genome of Capsaspora and the Origin of Animal Multicellularity. Cell.

[18] Lettice L.A., Devenney P., de Angelis C., Hill R.E. (2017). The Conserved Sonic Hedgehog Limb Enhancer Consists of Discrete Functional Elements that Regulate Precise Spatial Expression. Cell Rep..

[19] Long H.K., Osterwalder M., Welsh I.C., Hansen K., Davies J.O.J., Liu Y.E., Koska M., Adams A.T., Aho R., Arora N. (2020). Loss of Extreme Long-Range Enhancers in Human Neural Crest Drives a Craniofacial Disorder. Cell Stem Cell.

[20] Sikder S.K., Mitra D., Laurence J. (1994). Identification of a novel cell-type and context specific enhancer within the negative regulatory element of the human immunodeficiency virus type 1 long terminal repeat. Arch. Virol..

[21] MacKenzie A., Hing B., Davidson S. (2012). Exploring the effects of polymorphisms on cis-regulatory signal transduction response. Trends Mol. Med..

[22] Farley E.K., Olson K.M., Zhang W., Rokhsar D.S., Levine M.S. (2016). Syntax compensates for poor binding sites to encode tissue specificity of developmental enhancers. Proc. Natl. Acad. Sci. USA.

[23] Farley E.K., Olson K.M., Zhang W., Brandt A.J., Rokhsar D.S., Levine M.S. (2015). Suboptimization of developmental enhancers. Science.

[24] Farley E.K., Olson K.M., Levine M.S. (2015). Regulatory Principles Governing Tissue Specificity of Developmental Enhancers. Cold Spring Harb. Symp. Quant. Biol..

[25] Spurrell C.H., Dickel D.E., Visel A. (2016). The Ties That Bind: Mapping the Dynamic Enhancer-Promoter Interactome. Cell.

[26] Spitz F. (2016). Gene regulation at a distance: From remote enhancers to 3D regulatory ensembles. Semin. Cell Dev. Biol..

[27] Rada-Iglesias A., Grosveld F.G., Papantonis A. (2018). Forces driving the three-dimensional folding of eukaryotic genomes. Mol. Syst. Biol..

[28] Rowley M.J., Corces V.G. (2018). Organizational principles of 3D genome architecture. Nat. Rev. Genet..

[29] Williamson I., Kane L., Devenney P.S., Flyamer I.M., Anderson E., Kilanowski F., Hill R.E., Bickmore W.A., Lettice L.A. (2019). Developmentally regulated Shh expression is robust to TAD perturbations. Development.

[30] Hnisz D., Weintraub A.S., Day D.S., Valton A.L., Bak R.O., Li C.H., Goldmann J., Lajoie B.R., Fan Z.P., Sigova A.A. (2016). Activation of proto-oncogenes by disruption of chromosome neighborhoods. Science.

[31] Furlong E.E.M., Levine M. (2018). Developmental enhancers and chromosome topology. Science.

[32] Kong S., Bohl D., Li C., Tuan D. (1997). Transcription of the HS2 enhancer toward a cis-linked gene is independent of the orientation, position, and distance of the enhancer relative to the gene. Mol. Cell. Biol..

[33] Murakawa Y., Yoshihara M., Kawaji H., Nishikawa M., Zayed H., Suzuki H., Fantom C., Hayashizaki Y. (2016). Enhanced Identification of Transcriptional Enhancers Provides Mechanistic Insights into Diseases. Trends Genet..

[34] Creyghton M.P., Cheng A.W., Welstead G.G., Kooistra T., Carey B.W., Steine E.J., Hanna J., Lodato M.A., Frampton G.M., Sharp P.A. (2010). Histone H3K27ac separates active from poised enhancers and predicts developmental state. Proc. Natl. Acad. Sci. USA.

[35] Holmqvist P.H., Mannervik M. (2013). Genomic occupancy of the transcriptional co-activators p300 and CBP. Transcription.

[36] Stergachis A.B., Neph S., Reynolds A., Humbert R., Miller B., Paige S.L., Vernot B., Cheng J.B., Thurman R.E., Sandstrom R. (2013). Developmental fate and cellular maturity encoded in human regulatory DNA landscapes. Cell.

[37] Song M., Yang X., Ren X., Maliskova L., Li B., Jones I.R., Wang C., Jacob F., Wu K., Traglia M. (2019). Mapping cis-regulatory chromatin contacts in neural cells links neuropsychiatric disorder risk variants to target genes. Nat. Genet..

[38] Graur D., Zheng Y., Price N., Azevedo R.B., Zufall R.A., Elhaik E. (2013). On the immortality of television sets: “function” in the human genome according to the evolution-free gospel of ENCODE. Genome Biol. Evol..

[39] Villar D., Berthelot C., Aldridge S., Rayner T.F., Lukk M., Pignatelli M., Park T.J., Deaville R., Erichsen J.T., Jasinska A.J. (2015). Enhancer evolution across 20 mammalian species. Cell.

[40] Blow M.J., McCulley D.J., Li Z., Zhang T., Akiyama J.A., Holt A., Plajzer-Frick I., Shoukry M., Wright C., Chen F. (2010). ChIP-Seq identification of weakly conserved heart enhancers. Nat. Genet..

[41] May D., Blow M.J., Kaplan T., McCulley D.J., Jensen B.C., Akiyama J.A., Holt A., Plajzer-Frick I., Shoukry M., Wright C. (2011). Large-scale discovery of enhancers from human heart tissue. Nat. Genet..

[42] Oldridge D.A., Wood A.C., Weichert-Leahey N., Crimmins I., Sussman R., Winter C., McDaniel L.D., Diamond M., Hart L.S., Zhu S. (2015). Genetic predisposition to neuroblastoma mediated by a LMO1 super-enhancer polymorphism. Nature.

[43] Baggs J.E., Hayes K.R., Hogenesch J.B. (2005). Comparative genomics as a tool in the understanding of eukaryotic transcriptional regulation. Curr. Opin. Genet. Dev..

[44] Visel A., Bristow J., Pennacchio L.A. (2007). Enhancer identification through comparative genomics. Semin. Cell Dev. Biol..

[45] MacKenzie A., Purdie L., Davidson D., Collinson M., Hill R.E. (1997). Two enhancer domains control early aspects of the complex expression pattern of Msx1. Mech. Dev..

[46] Miller K.A., Barrow J., Collinson J.M., Davidson S., Lear M., Hill R.E., Mackenzie A. (2007). Ahighly conserved Wnt-dependent TCF4 binding site within the proximal enhancer of the anti-myogenic Msx1 gene supports expression within Pax3-expressing limb bud muscle precursor cells. Dev. Biol..

[47] Miller K.A., Davidson S., Liaros A., Barrow J., Lear M., Heine D., Hoppler S., MacKenzie A. (2008). Prediction and characterisation of a highly conserved, remote and cAMP responsive enhancer that regulates Msx1 gene expression in cardiac neural crest and outflow tract. Dev. Biol..

[48] Grice J., Noyvert B., Doglio L., Elgar G. (2015). A Simple Predictive Enhancer Syntax for Hindbrain Patterning Is Conserved in Vertebrate Genomes. PLoS ONE.

[49] Smith N.G., Brandstrom M., Ellegren H. (2004). Evidence for turnover of functional noncoding DNA in mammalian genome evolution. Genomics.

[50] Davidson S., Starkey A., MacKenzie A. (2009). Evidence of uneven selective pressure on different subsets of the conserved human genome; implications for the significance of intronic and intergenic DNA. BMC Genom..

[51] Hokfelt T., Barde S., Xu Z.D., Kuteeva E., Ruegg J., le Maitre E., Risling M., Kehr J., Ihnatko R., Theodorsson E. (2018). Neuropeptide and Small Transmitter Coexistence: Fundamental Studies and Relevance to Mental Illness. Front. Neural. Circuits.

[52] Alpar A., Benevento M., Romanov R.A., Hokfelt T., Harkany T. (2019). Hypothalamic cell diversity: Non-neuronal codes for long-distance volume transmission by neuropeptides. Curr. Opin. Neurobiol..

[53] Ueda H. (2006). Molecular mechanisms of neuropathic pain-phenotypic switch and initiation mechanisms. Pharmacol. Ther..

[54] Ebner K., Singewald N. (2006). The role of substance P in stress and anxiety responses. Amino Acids.

[55] Davidson S., Miller K.A., Dowell A., Gildea A., Mackenzie A. (2006). A remote and highly conserved enhancer supports amygdala specific expression of the gene encoding the anxiogenic neuropeptide substance-P. Mol. Psychiatry.

[56] Shanley L., Lear M., Davidson S., Ross R., MacKenzie A. (2011). Evidence for regulatory diversity and auto-regulation at the TAC1 locus in sensory neurones. J. Neuroinflammation..

[57] Shanley L., Davidson S., Lear M., Thotakura A.K., McEwan I.J., Ross R.A., MacKenzie A. (2010). Long-range regulatory synergy is required to allow control of the TAC1 locus by MEK/ERK signalling in sensory neurones. Neurosignals.

[58] Hay C.W., Shanley L., Davidson S., Cowie P., Lear M., McGuffin P., Riedel G., McEwan I.J., MacKenzie A. (2014). Functional effects of polymorphisms on glucocorticoid receptor modulation of human anxiogenic substance-P gene promoter activity in primary amygdala neurones. Psychoneuroendocrinology.

[59] Barson J.R., Morganstern I., Leibowitz S.F. (2010). Galanin and consummatory behavior: Special relationship with dietary fat, alcohol and circulating lipids. Exp. Suppl..

[60] Davidson S., Lear M., Shanley L., Hing B., Baizan-Edge A., Herwig A., Quinn J.P., Breen G., McGuffin P., Starkey A. (2011). Differential activity by polymorphic variants of a remote enhancer that supports galanin expression in the hypothalamus and amygdala: Implications for obesity, depression and alcoholism. Neuropsychopharmacology.

[61] McEwan A.R., Davidson C., Hay E., Turnbull Y., Erickson J.C., Marini P., Wilson D., McIntosh A.M., Adams M.J., Murgatroyd C. (2020). CRISPR disruption and UK Biobank analysis of a highly conserved polymorphic enhancer suggests a role in male anxiety and ethanol intake. Mol. Psychiatry.

[62] Low B.E., Christianson G.J., Lowell E., Qin W., Wiles M.V. (2020). Functional humanization of immunoglobulin heavy constant gamma 1 Fc domain human FCGRT transgenic mice. mAbs.

[63] Pi M., Xu F., Ye R., Nishimoto S.K., Kesterson R.A., Williams R.W., Lu L., Quarles L.D. (2020). Humanized GPRC6A(KGKY) is a gain-of-function polymorphism in mice. Sci. Rep..

[64] Gu B., Posfai E., Gertsenstein M., Rossant J. (2020). Efficient Generation of Large-Fragment Knock-In Mouse Models Using 2-Cell (2C)-Homologous Recombination (HR)-CRISPR. Curr. Protoc. Mouse. Biol..

[65] Cheah S.S., Behringer R.R. (2000). Gene-targeting strategies. Methods Mol. Biol..

[66] Hodgson K., Mufti S.J., Uher R., McGuffin P. (2012). Genome-wide approaches to antidepressant treatment: Working towards understanding and predicting response. Genome Med..

[67] Budney A.J., Sofis M.J., Borodovsky J.T. (2019). An update on cannabis use disorder with comment on the impact of policy related to therapeutic and recreational cannabis use. Eur. Arch. Psychiatry Clin. Neurosci..

[68] Nicoll G., Davidson S., Shanley L., Hing B., Lear M., McGuffin P., Ross R., MacKenzie A. (2012). Allele-specific differences in activity of a novel cannabinoid receptor 1 (CNR1) gene intronic enhancer in hypothalamus, dorsal root ganglia, and hippocampus. J. Biol. Chem..

[69] Hay E.A., Cowie P., McEwan A.R., Ross R., Pertwee R.G., MacKenzie A. (2020). Disease-associated polymorphisms within the conserved ECR1 enhancer differentially regulate the tissue-specific activity of the cannabinoid-1 receptor gene promoter; implications for cannabinoid pharmacogenetics. Hum. Mutat..

[70] Hing B., Davidson S., Lear M., Breen G., Quinn J., McGuffin P., MacKenzie A. (2012). A polymorphism associated with depressive disorders differentially regulates brain derived neurotrophic factor promoter IV activity. Biol. Psychiatry.

[71] Burton M.A., Lillycrop K.A. (2019). Nutritional modulation of the epigenome and its implication for future health. Proc. Nutr. Soc..

[72] Doherty T.S., Roth T.L. (2018). Epigenetic Landscapes of the Adversity-Exposed Brain. Prog. Mol. Biol. Transl. Sci..

[73] Kader F., Ghai M., Maharaj L. (2018). The effects of DNA methylation on human psychology. Behav. Brain. Res..

[74] Reul J.M. (2014). Making memories of stressful events: A journey along epigenetic, gene transcription, and signaling pathways. Front. Psychiatry.

[75] Murgatroyd C., Wu Y., Bockmuhl Y., Spengler D. (2010). The Janus face of DNA methylation in aging. Aging.

[76] Murgatroyd C., Wu Y., Bockmuhl Y., Spengler D. (2010). Genes learn from stress: How infantile trauma programs us for depression. Epigenetics.

[77] Murgatroyd C., Patchev A.V., Wu Y., Micale V., Bockmuhl Y., Fischer D., Holsboer F., Wotjak C.T., Almeida O.F., Spengler D. (2009). Dynamic DNA methylation programs persistent adverse effects of early-life stress. Nat. Neurosci..

[78] Hay E.A., McEwan A., Wilson D., Barrett P., D’Agostino G., Pertwee R.G., MacKenzie A. (2019). Disruption of an enhancer associated with addictive behaviour within the cannabinoid receptor-1 gene suggests a possible role in alcohol intake, cannabinoid response and anxiety-related behaviour. Psychoneuroendocrinology.

[79] Hay E.H., Cowie P., McEwan A.J., Wilson D., Ross R., Barrett P., Pertwee R.G., MacKenzie A. (2019). Genetic and pharmacological influences modulating tissue specific regulation of the cannabinoid receptor-1 (CB1); implications for cannabinoid pharmacogenetics. BioRxiv.

[80] Visscher P.M., Wray N.R., Zhang Q., Sklar P., McCarthy M.I., Brown M.A., Yang J. (2017). 10 Years of GWAS Discovery: Biology, Function, and Translation. Am. J. Hum. Genet..

[81] King C.R., Nicolae D.L. (2014). GWAS to Sequencing: Divergence in Study Design and Analysis. Genes.

[82] Consortium G.T. (2015). Human genomics. The Genotype-Tissue Expression (GTEx) pilot analysis: Multitissue gene regulation in humans. Science.

[83] O’Brien H.E., Hannon E., Hill M.J., Toste C.C., Robertson M.J., Morgan J.E., McLaughlin G., Lewis C.M., Schalkwyk L.C., Hall L.S. (2018). Expression quantitative trait loci in the developing human brain and their enrichment in neuropsychiatric disorders. Genome Biol..

